# Comparing the use of conventional and three-dimensional printing (3DP) in mandibular reconstruction

**DOI:** 10.1186/s12938-022-00989-6

**Published:** 2022-03-19

**Authors:** Ailis Truscott, Reza Zamani, Mohammad Akrami

**Affiliations:** 1 Medical School, College of Medicine and Health, Exeter, UK; 2 Department of Engineering, College of Engineering, Mathematics, and Physical Sciences, University of Exeter, Exeter, EX4 4QF UK

**Keywords:** Mandibular reconstruction, Mandible, Reconstructive surgery, 3D-printing, Conventional surgery

## Abstract

**Background:**

There are a number of clinical disorders that require mandibular reconstruction (MR). Novel three-dimensional (3D) printing technology enables reconstructions to be more accurate and beneficial to the patient. However, there is currently no evidence identifying which techniques are better suited for MR, based on the type of clinical disorder the patient has. In this study, we aim to compare 3D techniques with conventional techniques to identify how best to reconstruct the mandible based on the clinical cause that necessitates the reconstructive procedure: cancerous or benign tumours, clinical disorders, infection or disease and trauma or injury.

**Methods:**

PubMed, Scopus, Embase and Medline were searched to identify relevant papers that outline the clinical differences between 3D and conventional techniques in MR. Data were evaluated to provide a clear outline of suitable techniques for surgery.

**Results:**

20 of 2749 papers met inclusion criteria. These papers were grouped based on the clinical causes that required MR into four categories: malignant or benign tumour resection; mandibular trauma/injury and other clinical disorders.

**Conclusions:**

The majority of researchers favoured 3D techniques in MR. However, due to a lack of standardised reporting in these studies it was not possible to determine which specific techniques were better for which clinical presentations.

## Background

Mandibular defects that require reconstruction can be caused by a variety of disorders [1, 2]. Malignant or benign tumour removal can require resection, disorders of the jaw (mainly osteoradionecrosis or osteonecrosis, types of bone death) can impair functionality, disease or infection (osteomyelitis) can develop in the bone, and injury can cause fractures [3]. These can all lead to a restricted quality of life as both function and aesthetics may be impaired [4]. Jewer et al. [5] described a method of classification for these defects based upon whether they affected the central or lateral areas of the mandible, as this affects the complex process of reconstruction. While central defects require multiple osteotomies, lateral defects can be reconstructed using a single straight bone [3]. The gold standard of treatment is to restore both aesthetics and function, including mastication, speech and swallowing, and to allow accessible airways [3, 6].

Autologous bone grafts fixed with titanium plates is the standard method of MR [7], this involves transport of bone from a donor site, to a recipient site, all on the same patient. There are two main surgical techniques that can be used; vascularised and non-vascularised bone grafts [3]. Most frequently used are vascular free flaps, which contain an intrinsic blood supply, reducing recovery time and allowing healing independently from the damaged recipient site [3, 6]. Secondary reconstructions as a result of radiation, infection or previous surgical intervention, use this technique most often [3]. The most prevalent vascularised graft is the fibula free flap (FFF) [7–9], introduced by Hidalgo in 1989 [10]. The size and shape of the fibula allows FFF reconstructions to have low donor site morbidity, large diameter blood vessels and the flexibility to shape the bone to follow the contour of the native mandible [11]. Other common vascularised flap techniques include the deep circumflex iliac artery (DCIA), whereby a section of the iliac bone is harvested along with a section of the circumflex iliac artery, introduced by Taylor et al. [12] and the scapular osteocutaneous free flap (SOFF), introduced by Swartz et al. [13]. Using multiple osteotomies, all have shown the ability to reproduce the native mandibular contour [14], especially in defects larger than 6 cm [15, 16].

Non-vascularised grafts, such as iliac bone or costochondral rib, are less reported in the literature. They are used for smaller defects where the soft tissue is not affected; most commonly found after trauma [3]. It has been observed that the lack of intrinsic blood supply limits the healing process, increasing the risk of infection and postoperative complications [15, 16]. Furthermore, in the treatment of malignant tumours, radiation therapy leaves these flaps increasingly prone to osteoradionecrosis [17]. Given the increased rates of preparatory and postoperative complexities and overall increased procedure time, seen in conventional MR, new techniques using new technologies have been developed to address these issues.

Since its first use by Hirsch et al. [14] in 2009, the use of 3D printing techniques in MR surgery has become more prevalent throughout the literature [18]. 3D printing, also known as rapid prototyping (RP) or additive manufacturing (AM), will often use computer-aided design and computer-aided manufacturing (CAD/CAM) to produce the 3D models or tools designed to improve reconstruction; this technique uses multiple two-dimensional computed tomography (CT) scans of the mandibular area. These scans are saved as Digital Imaging and Communications in Medicine (DICOM) files [14, 18, 19] and converted to 3D models in a Standard Tessellation Language (STL) format. This 3D model is then printed layer-by-layer using one of seven 3D printing technologies [20, 21]. Over the last decade, this technology has been made more publicly available, with hospitals themselves developing multidisciplinary workflows [22]. Bolzoni et al. [23] performed a cost analysis between patients treated with CAM/CAD technology and patients treated conventionally, finding that the overall health care cost of these treatment plans was comparable. Minimal cost difference and increased accessibility have allowed for the development of a wide range of applications [24]. Not only can 3D models be used for a preoperative surgical ‘dry-run’ [25, 26], but their morphology can be used to preoperatively bend titanium reconstruction plates around the neo-mandible, as opposed to contouring plates intraoperatively [27, 28]. Alternatively, titanium plates can be personalised and 3D printed themselves [29, 30], and occlusal splints and dental implants can also be prototyped [31, 32]. CAD/CAM allows for possibilities not available using conventional techniques; for example, the ability to mirror the unaffected mandible, allowing better mandibular symmetry, the ability to plan osteotomy measurements for resection and the possibility to restore appropriate occlusion [14, 33]. These technologies reduce reliance on an individual surgeon’s expertise. A variety of outcomes have been described in the literature when observing CAD/CAM techniques: operative time [34–36], thus ischaemic time [37, 38], accuracy [39, 40], postoperative complications [41], aesthetics [42] and cost [43, 44].

Nevertheless, despite the vast amount of 3D applications available and the wide variety of outcomes observed using these techniques, there is no research comparing 3D techniques and conventional techniques in MR when looking at the treatment for a specific clinical cause. In this review, we aim to compare CAD/CAM techniques with conventional techniques to identify how best to reconstruct the mandible based on the clinical cause that necessitates it.

## Results

### Characteristics of included studies

Of the 20 studies included, the patient characteristics reported were grouped into four categories: malignant or benign tumour removal (*n* = 18), clinical disorders (*n* = 9), disease/infection (*n* = 4), injury or trauma (*n* = 4). Two studies included patients with undetermined diagnoses, referred to as ‘other’ (Table 3). Within these papers, various CAD/CAM techniques were used: 3D models for preoperative ‘dry-run’ surgery (*n* = 4), preoperative bending of titanium plates using 3D models (*n* = 12), the manufacturing of patient-specific osteotomy cutting/resection guides (*n* = 11), 3D printed titanium reconstruction plates (*n* = 5) and printed splints for fracture reduction (*n* = 1) (Table 2).

### Clinical characteristics of included patients (Table 3)

Most researchers in this area disregard the clinical condition that necessitates MR and include patients with a variety of different disorders in the same group. For example, one study included patients, in the same cohort, with various cancerous tumours, osteoradionecrosis and even bilateral joint ankylosis and yet the patients treatment plan was the same, regardless of their different conditions [46]. This leads to overlap in the findings when identifying which techniques are better suited to MR for the treatment of specific clinical disorders.

The majority of research teams (*n* = 17; 85%) included patients with any form of malignant tumour. The most common cancer identified is squamous cell carcinoma (*n* = 14), then sarcoma (*n* = 11), any other form of carcinoma (*n* = 4), and cancer of unknown primary (*n* = 2). Similarly, patients with benign tumours also make up a large proportion of the patients treated for MR (*n* = 16; 80%). Ameloblastoma (*n* = 15), odontogenic keratocysts (*n* = 7), granulomas (*n* = 1), fibroma (*n* = 2), giant cell tumours (*n* = 1), osteochondroma (*n* = 1), odontogenic myxoma (*n* = 1), unspecified benign tumour (*n* = 1). Just under half the researchers refer to patients with some sort of disorder. For example, osteoradionecrosis (*n* = 8), osteonecrosis (*n* = 3), pseudoarthrosis, acquired deformity, joint ankylosis, osteoarthrosis, fibrous dysplasia and condylar reabsorption (*n* = 1 each). Four of the 20 studies, included in our review, refer to patients with some kind of infection or disease requiring MR, namely chronic or acute osteomyelitis [27, 42, 46, 47].

Only four referred to patients who required MR as a result of trauma and injury [26, 41, 42, 48]. King et al. [26] and Ramanathan et al. [41] were the only group to focus solely on patients with injury, specifically mandibular fractures, whilst Modabber et al. [42] and Modabber et al. [48] included these patients as examples within their larger cohorts (gunshot wound *n* = 1 and midfacial projectiles *n* = 3, respectively) (Table 3).

### Observed outcomes

There is no standardised procedure for reporting outcomes throughout the literature. Of the 20 papers included, 12 different outcomes were observed and grouped into five categories, all using various manufacturing techniques: operative/ischaemic time, accuracy, complications, aesthetics and cost (summary in Table 4). The most common outcomes observed was operative/ischaemic time (*n* = 11; 55%). The average number of outcomes observed per study was 1.95.

### Operative/ischaemic time

11 of the 20 research teams included, use operative time as a measurement to compare CAM/CAD and conventional MR surgery [25–27, 42, 44, 46–51]. 10 of these found that using CAM/CAD technology can reduce operative and therefore reduce the risk of extra complications. Only Yang et al. [51] found no significant difference between the surgical techniques. De Farias et al. [25] (*p* < 0.001), Gil et al. [47] (*p* < 0.04), Mahendru et al. [50] (*p* < 0.0001), Zhang et al. [46] (*p* < 0.05) and Tarsitano et al. [44] (*p* < 0.041) all found a statistically significant reduction in operative time in the 3D group compared to the control. Additionally, King et al. [26] found the mean operative time was 22.8 ± 2.1 min in the group treated conventionally and only 6.9 ± 0.3 min in the group treated using CAM/CAD technology (*p* < 0.0001).

On the other hand, Ayoub et al. [27] found that although the ischaemic time (*p* < 0.005) and reconstruction time (*p* < 0.001) were significantly shorter in the 3D group, the time required for sawing and shaping the flap was much longer than in the conventional group (*p* < 0.005). Therefore, along with Liu et al. [49] both studies found that although intraoperative time was reduced, the extra time for preoperative planning and shaping the transplant was increased, so there was no overall time gain. Yang et al. [51] also found no significant difference between the groups. Modabber et al. [48] found ischaemic time was significantly shorter in the 3D group (*p* < 0.014), with Modabber et al. [42] suggesting on average 15.6 min were saved.

### Accuracy

‘Accuracy’ was defined differently by different authors; in general, accuracy was quantified as a comparison of various length or angular measurements around the mandible. Depending on what measurements were being compared, ‘accuracy’ was therefore grouped into three broad categories: comparing the amount of bone harvested vs. bone used; comparing postoperative bilateral measurements; comparing pre- and post-operative measurements. 17 (85%) of the included 20 research papers referred to some form of accuracy as an outcome, with the majority of comparisons significantly suggesting that using 3D techniques allows more accurate reconstructions.

### Bone harvested vs. bone used

Four researchers referred to the ratio of bone harvested and bone used, all favouring the use of 3D techniques to reduce complications and wasted bone. In the conventional group Modabber et al. [42] and De Farias et al. [25] found the amount of bone harvested was often more than required, when compared to the group using CAM/CAD technology. This was found to be statistically significant by Modabber et al. [48] (3D: 0 cm excess versus Control: 1.92 cm average excess; *p* < 0.001). Ayoub et al. [27] found that in the 3D group there was no significant difference between the amount of bone harvested at the donor site and the amount of bone needed at the mandibular recipient site (87.6 ± 16.5 mm); and when compared to the conventional group, the average amount of bone harvested significantly exceeded the amount of bone required at the recipient site by 16.8 mm (± 5.6 mm; *p* < 0.001).

### Postoperative bilateral measurements

Six research teams refer to measurements taken postoperatively, with a trend to favour the use of CAM/CAD techniques. Lower differences in the bilateral condylar positions were found in the 3D group [52, 53], showing that 3D techniques allow better condylar symmetry. Yang et al. [51] found that 3D printed patient-specific plates significantly increased the accuracy of the reconstruction as the deviation of the bilateral mandibular angle was significantly lower than that of the group treated conventionally (*p* < 0.005). Naros et al. [52], Azuma et al. [28] and Bartier et al. [39] found that sagittal mandibular angle symmetry was improved in the 3D group (*p* < 0.005, *p* < 0.05 and *p* < 0.034, respectively), but Bartier et al. [39] went on to find no significance in the difference in the axial or coronal mandibular angle between the two groups. Furthermore, Zhang et al. [46] found that there was no significant condylar deviation bilaterally in the 3D group, allowing good aesthetic symmetry, although this outcome was not compared to the control group. Wurm et al. [53] also found significantly better plate fitting accuracy in the 3D group (*p* < 0.048), allowing for better mandibular contour.

### Pre- and postoperative measurements

Of the 17 research teams that report on the accuracy, seven of these compare various measurements taken pre- and postoperatively. Whilst some measurements would indicate 3D techniques can be more accurate, multiple groups reported non-significant results for different measurements. Bartier et al. [39] and De Maesschalck et al. [30] compared CT scans for various linear and angular measurements on the affected mandibular side, pre- and postoperatively. Bartier et al. [39] found that deviation of the coronal mandibular angle (*p* < 0.019) and deviation of the mandibular ramus (*p* < 0.006) and mandibular body height (*p* < 0.014) were significantly lower in the 3D group. However, there was no significant difference between intercondylar distance and axial or sagittal mandibular angles, between groups treated conventionally and groups treated using CAM/CAD technology. Two researchers contradicted this, finding a significant difference in the sagittal mandibular angle, between the two groups, favouring the 3D group (*p* < 0.034) and (*p* < 0.005) [40, 51]. De Maesschalck et al. [30] found no statistical difference between any linear or angular measurements between the control and test groups, showing that both techniques can provide equal accuracy. Ciocca et al. [29] compared the lateral and vertical shift (deviation) pre- and postoperatively in reference to the native mandibular contour, between both groups as a measure of symmetry. There was no statistical significance in any deviation, indicating that both 3D and conventional techniques are suitable for accurate reconstructions. On the other hand, Tarsitano et al. [40] observed bigonial diameter as a measure of lateral shift and also chin protrusion, finding significant differences that indicate 3D techniques can provide more accurate reconstructions, (*p* < 0.041 and *p* < 0.05, respectively). Bigonial distance deviation, favouring 3D techniques, were also found significant by Yang et al.’s [51] (*p* < 0.005) and Ayoub et al. [27] (1.3 mm ± 0.2 mm deviations versus 5.5 mm ± 2.5 mm deviations; *p* < 0.001). There was, however, no significant deviation in the midline difference between the groups [40]. Yang et al. [51] observed the deviation in bilateral condylar position and condylar angle, pre- and postoperatively between the groups, finding no significant difference. Opposing this, Zhang et al. [54] found significant data that proved that 3D techniques can reduce condylar position deviation (*p* < 0.026) and mandible contour symmetry (*p* < 0.01).

### Complications

Only a quarter of researchers (*n* = 5; 25%) included in this review, compared the frequency of any postoperative complications between groups treated conventionally and groups treated using 3D technology. All report that using 3D techniques reduce the frequency of postoperative complications. Zhang et al. [54] observed a higher rate of postoperative complications in the conventionally treated group, but this was not proven to be significant. Gil et al. [47] found statistically significant differences in the frequency of titanium plate exposure (*p* = 0.009) and dental malocclusion (*p* = 0.03), with less incidence in the 3D group. Mahendru et al. [50] found no flap failures in the CAM/CAD-treated group, with two incidences found in the group treated conventionally. Furthermore, whilst only one patient in the CAM/CAD group required re-exploration, seven patients underwent this additional procedure in the conventional group. Finally, malocclusion was reported in only 2.5% of patients in the 3D treated group, and 15% of patients in the conventionally treated group. On the other hand, whilst there was no incidence of plate fractures in the conventional control group, one incidence was reported in the 3D group, there was however, no explanation of what type of error led to this plate fracture. Tarsitano et al. [44] observed a 10% rate of flap failure, as the transplant was rejected, in the group treated conventionally, compared to the 3D group. Ramanathan et al. [41] observed malocclusion as a complication when treating patients with mandibular fractures. Dental malocclusion was corrected in 13 of the 15 patients in the group treated conventionally and all 15 patients in the 3D group (*p* = 0.48). Interfragmentary separation postoperatively in the 3D group was 0.00 mm and 0.47 mm in the group treated conventionally (*p* = 0.001).

### Aesthetics

Despite being a key aspect of Quality of Life, only three researchers observed the final aesthetic result as an outcome for MR. All research teams that observed aesthetics as an outcome favoured 3D techniques, providing patients with a more symmetrical and better postoperative appearance. Modabber et al. [42] found that patient self-assessment for aesthetics was graded higher in the 3D group (3D: avg 88.5 Control: avg 67.9). Mahendru et al. [50] graded aesthetics out of 5 (3D: average = 3.64 versus Control: average = 2.55). De Farias et al. [25] had both medical staff and non-medical associates of the patient grade the aesthetic outcome, in a rank out of 10 (3D: Medical average = 7.7 Non-medical average = 9.2 Control: Medical average = 6.0 Non-medical average = 8.8). Ramanathan et al. [41] also observed patient-based outcomes through grading of patient comfort, however did not look specifically at any final aesthetic results. They found the 3D group graded comfort much higher (0.20) than the control group (8.20; *p* < 0.001); furthermore, surgeon comfort was significantly higher in the 3D group [41].

### Costs

Only five researchers specifically compared the cost of the two techniques; its reported that CAM/CAD technology can be comparable in price to conventional techniques. Ayoub et al. [27] suggested that although the immediate cost of the CAM/CAD-assisted surgery was much greater, this would even out over the long term due to the lower incidents of postoperative complications [27] and the reduction in operative time [44]. Tarsitano et al. [44] performed a cost analysis identifying that in Italy the institutional cost per minute of treatment was €30 while the total cost of 3D-assisted surgery was €3500 (€500 for conventional surgery). However, the shorter operative time and hospital stay saves the patient €3450, so the cost is balanced out. The cost of 3D-printing technology is, however, frequently referred to as very expensive [29, 51]. King et al. [26] found the overall operating costs were $2306.45 ± 212.44 for those treated using conventional techniques and these were significantly lower in the group treated using CAM/CAD technology, with an estimate of only $698.00 ± 30.35 (*p* < 0.0001).

## Discussion

The aim of this systematic review is to evaluate the use of CAD/CAM techniques in MR, when compared to conventional techniques in an attempt to identify which techniques are best suited to treat different clinical conditions. Of the 20 studies included, the reported outcomes are very heterogeneous (see Table 4). It was found that mandibular fracture reconstruction studies were the only reports where the cause of the disorder was identified clearly and patients were grouped based on their specific clinical disorder [26, 41]; the majority of research teams grouped patients together who needed reconstruction, regardless of their specific clinical cause. This lack of a standardised procedure makes it impossible to determine if 3D printing techniques are more effective in mandibular reconstruction when compared to conventional techniques; and more specifically, which of the various 3D techniques would be most suitable for the treatment of different clinical disorders [29]. Several studies have also criticised this lack of standardised reporting [18, 25, 30, 33, 41, 51, 55]. This could be as a result of how modern this technology is, with several research teams stating that they are the first of their kind using these techniques [27, 46, 51, 52]. The lack of standardisation means outcomes are being defined and evaluated differently between research groups, making it difficult to draw comparisons and/overall conclusions from the literature [18]. Louvrier et al. [24] conducted a review in 2017, finding a majority (74%) of published studies in this area were non-comparative retrospective case reports or case studies with 10 patients or fewer. This could be because, before 3D printing was made more accessible, it was limited to use in complex cases, where conventional surgery was not an option [21]. Although benefits of this technology are often described [21], this area of research is lacking in prospective comparative studies that clearly define which of these novel techniques is best suited for MR based on the type of clinical cause that necessitates it. As grouping of results based on clinical cause proved to be impossible at this stage, due to the mass overlap in these studies, we discuss the studies based on outcomes observed (see Table 4).

### Operative/ischaemic time

In the 20 studies included, the most reported outcome, was operative/ischaemic time, of which 11 (55%) used this as a clinical outcome [25–27, 42, 44, 46–51]. This agreed with another systematic review by Serrano et al. [21] Of these 11 studies, 10 included patients treated for tumour removal, five for various disorders, four for any disease/infection and three for injury/trauma (Tables 3 and 4). Only one did not favour the 3D protocol, Yang et al. [51] found no significant difference in operative time. However, in a previous study by some of the same authors, the 3D techniques did save time (707 min vs. 534 min; *p* < 0.0003) [56], so this study may not be representative, especially considering the small sample size of only 33 patients total. An important factor affecting operative time is the complexity of the reconstruction. This needs to be accounted for between the two groups when comparing the results. Seven of these researchers made no reference to the characteristics of each patient group [25, 26, 42, 44, 46, 48, 49]. Tarsitano et al. [44] identified this weakness, stating that it causes potential bias, as findings may result from confounding variables and not the technique used. One example is Mahendru et al. [50], who found that the number of osteotomies required between the two group was significantly different. Although they stated a significant reduction in operative time in the 3D group, this could have simply been because the surgeries were less complex to begin with. Ayoub et al. [27] and Gil et al. [47] found a significant reduction in operative time in the 3D group and found no significant differences in the complexity of the reconstructions between each group; the findings are therefore more likely representative of the true benefits of using 3D techniques.

### Accuracy

Seventeen of the 20 reports measure some sort of accuracy of the reconstruction as an outcome (Table 4). The issue is that accuracy is not clearly defined in the literature, so every researcher uses different measurements to assess this. From the studies included, three different accuracy categories were grouped: bone harvested vs. bone used, postoperative bilateral measurements and comparing pre- and postoperative measurements of various angles and lengths.

### Bone harvested vs. bone used

The size of the graft has been associated with the frequency of postoperative complications, and so harvesting only the necessary amount of bone is essential [57]. Four research teams observed this as an outcome [25, 27, 42, 48]. All four research teams included patients treated for tumour removal, two for various disorders and infections and one included patients requiring reconstruction as a result of injury—see summary in Table 3. All four researchers favoured 3D-printing techniques to reduce the amount of excess bone harvested. Ayoub et al. [27] and Modabber et al. [48] found statistical significance. De Farias et al. [25] and Modabber et al. [42] did not use any statistical testing, so their results do not have the power to standardise their methods of 3D-printing. One of the first major studies in this area corroborates these findings; Hanasono and Skoracki [34] also found better anatomic accommodation of the bone flap in the 3D group. They used virtual planning and 3D printing to create cutting guides, which allowed more accurate harvesting of the fibula flap; this therefore simplified the process of osteotomy creation as the flap size was already shaped to best suit the anatomy of the mandible.

### Postoperative bilateral measurements

Bilateral mandibular angles have been used as a measurement to assess accuracy and symmetry [28, 39, 51, 52], but the intercondylar position can also be used [46, 52, 53]. Six studies used postoperative bilateral measurements as a measure of accuracy [28, 39, 46, 51–53]. All six researchers included patients treated for tumour removal, five involved patients with various disorders and one with infections (Table 3). Even within this category, there is heterogeneity between the methods used. Choi et al. [58] stated that choosing the location to measure was difficult, which is why there is such variation in the literature. Furthermore, Zhang et al. [46] is the only study not to compare these measurements to the patients that were treated conventionally. To make these measurements, Azuma et al. [28] used pantomography. Although this is a common method for symmetry evaluation [59], it has been criticised because it does not account for differences in body size. Accuracy was also defined by Wurm et al. [53] as the plate–bone contact ratio, as this would allow a more natural mandible contour. An increased gap between the donor bone and the reconstruction plate (the titanium plate used to fix the transplanted bone in place) may increase the risk of infection [28], so this measurement of accuracy is also an important factor in postoperative complications.

### Pre- and postoperative measurements

Seven research teams defined accuracy by comparing pre- and postoperative measurements of various angles and lengths on the mandible [27, 29, 30, 39, 40, 51, 54]; see Table 3. In the literature there are large inconsistencies in these results and heterogeneity regarding how they are measured [21]. Three techniques have been used: (1) comparing patients’ CT scans pre- and postoperatively [27, 39, 40, 54, 60, 61]; (2) comparing the postoperative CT scan with the DICOM models [29, 33, 51, 62]; (3) comparing CT scans with the prototype model [19, 30, 63]. CT scan resolution depends on access to good quality machines, this creates inequalities depending on the access to these machines as this will vary across countries depending on the money available in their healthcare systems [49]. Although resolution can be manually edited, working layer-by-layer is time consuming and subject to human error [64]. The quality of the CT scans will also affect the accuracy of any 3D printed models [65]. It has been suggested that postoperative results will never fully match preoperative plans, due to the additive effect of these human inaccuracies [58, 66]. This brings into question the relevance of these measurements when assessing accuracy. Three of these researchers refer to a limitation, in that because there is a strict preoperative planning process, if there are any changes intraoperatively, the 3D technique cannot adapt and the reconstruction relies, again, on the expertise of the surgeon [27, 29, 30]. On the other hand, this clear surgical plan can be utilised by less experienced surgeons, allowing them to complete more complex reconstructions earlier in their careers [26, 29].

### Complications

Five research teams reported on the incidence of postoperative complications [41, 44, 47, 50, 54] (Table 4). Although not always reported by the researchers included in this review, Chaine et al. [67] analysed the complications following FFF reconstructions, finding common complications to include: flap loss, malocclusion, soft tissue necrosis, fistula growth, donor site morbidity and even facial asymmetry. Four of these five research teams included patients requiring reconstruction as part of the treatment for tumour removal, whilst Gil et al. [47] also included patients being treated for osteoradionecrosis disorder and osteomyelitis infections; Ramanathan et al. [41] reported on the incidence of complication in the treatment of patients with mandibular injuries (Table 3). Despite the various complications observed, all researchers favoured the 3D protocol, which allows for the lowest incidence of any postoperative complications. This was found to be significant by Gil et al. [47]. Complications are an important factor as they will affect the overall cost for the patient [44]. Furthermore, there is a link between ischaemic (operative) time and the survival rate of graft, and so lower incidence of complications may be as a result of shorter ischaemic time [68]. An additional limitation of the literature is the heterogeneous length of follow-up time. 11 of the 20 researchers included do not refer to any time frame for their follow-up, with the researchers that do follow up having vast inconsistencies in the length of time they waited to check in on their patients. The lack of follow-up reported could be because negative findings are often reported as an absence of results by the researchers; most published results are positive and therefore the overall literature may be biased [69].

### Aesthetics

Only three research teams observed aesthetics as an outcome (Table 4). All three looked at reconstruction aesthetics after tumour removal [25, 50], with Modabber et al. [42] also including patients treated for various disorders, infections and injuries (Table 3). Although no researchers used statistical analysis, they all observed better aesthetics in the 3D group. A major limitation of the literature is the lack of reported outcomes that focus on how the patient has perceived the success of the surgery, not merely the report of the researchers. Only one study used patients’ self-assessment [42]. Furthermore, no research teams included in this review observed any patient Quality of Life assessment [21]. This is a major aspect of reconstructive surgery, and yet is so infrequently observed in the literature, as shown by the lack of reporting by the researchers included in our review. This may be due to the only recent development of these studies to include larger cohorts of patients, with Bartier et al. [39] and Gil et al. [47] stating that this outcome should be observed in future research.

### Cost

Although only five research teams use cost as an observed outcome in their own studies [26, 27, 29, 44, 51] (Table 4), 15 of the 20 research teams refer to cost in their discussion. This area is of great controversy in the literature. Seven of these researchers state that 3D printing technology is more costly than conventional methods, with regard to the entire surgical process, including the preoperative planning and printing of the 3D components and the hospital stay required by the patients [27, 29, 39, 49, 51, 52, 54]. On the other hand, De Maesschalck et al. [30] and King et al. [26] suggest that 3D printing is overall a cheaper alternative to conventional surgery, and less costly to the patient. Additionally, it has been stated that although 3D printing is more expensive, the time saved during surgery and the length of hospital stay balances out this extra cost [25, 41, 44, 47, 48, 50]. It was also reported that operative time usually accounts for 30–40% of the total cost of reconstruction [70]. Tarsitano et al. [44] is the only team to conduct a full cost analysis and identified that the institutional cost per minute for MR was €30 in Italy; this accounts for the resources used, the length of stay in the hospital and any specialist CAM/CAD equipment. Other researchers in the literature found that in Switzerland the cost per minute is $47.50 [43] and in New York $103 [71]. Therefore, the differences reported in the literature could be as a result of the varying costs in different countries, and future research would be needed to investigate this. Additionally, with 3D printers becoming more commercially available, an in-house workflow has been described to reduce time and cost for the patient when compared to using external printing companies [26, 52].

## Limitations

One limitation is that we only searched the literature published in English. This may have introduced bias to our findings. Despite this, many studies we identified are published from non-English speaking countries, and so our results may still be representative. Additionally, we can assume that non-published research would have a lower quality of evidence, so would not meet our criteria anyway.

## Conclusions

This is the first review to observe MR based on the clinical cause that necessitates it, comparing which techniques provide better clinical outcomes: conventional or CAD/CAM. The current literature in this area is limited by the lack of a standardised procedure, variations in clinical presentations both in degrees or the types of pathology, so it proved difficult to draw decisive conclusions. We found we were unable to determine which specific techniques work best for reconstructions for specific clinical causes due to the overlap of various clinical conditions within the same study group. Future research needs to become more standardised, grouping or reporting patients based on the type of clinical disorder they have, so it can be evaluated how best to treat similar patients in the future. Furthermore, there needs to be a procedure for reporting the outcomes of these studies, so that similar systematic reviews can be repeated in the future and provide more conclusive results. Despite this, we were able to accumulate literature that describes the clinical benefits of using CAD/CAM techniques over conventional methods for MR. Of all the outcomes observed, CAD/CAM techniques were favoured in the majority of studies. This indicates that despite the clinical cause, CAD/CAM should be favoured by surgeons for MR, as this allows shorter operative time, better accuracy and aesthetics, fewer complications and can be completed at an equal cost to conventional methods.

## Materials and methods

### Search strategy

**Fig. 1:**
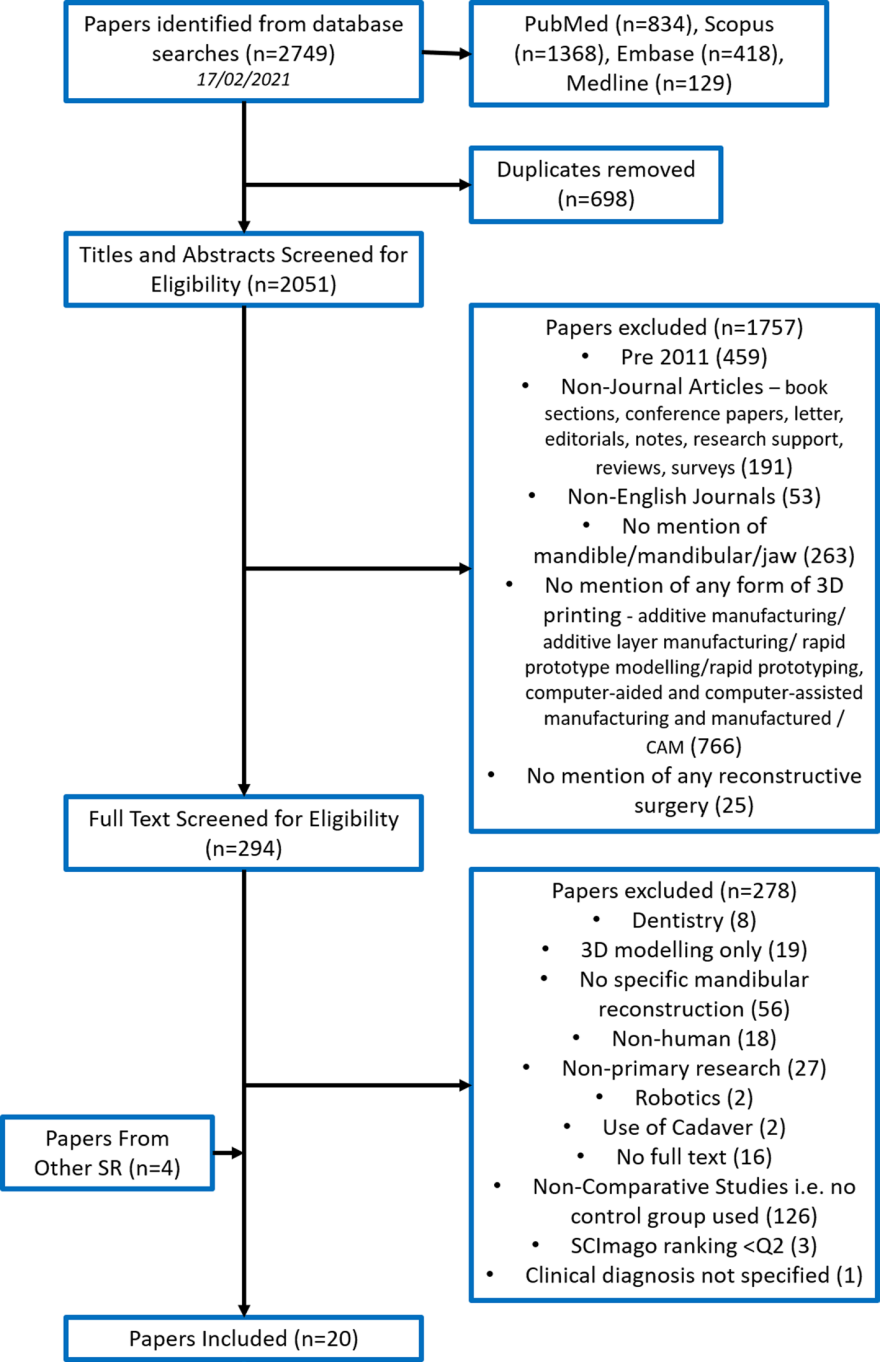
PRISMA flow diagram of search strategy. A flow diagram of our study based on the preferred reporting items for systematic review and meta-analyses (PRISMA) method

The Population, Intervention, Comparison, Outcome (PICO) search strategy was used to identify studies in PubMed, Scopus, Embase and Medline; the latter two accessed through Ovid. The following keywords were used:

1. ‘Mandibular reconstruction’—all variations of both words were used, combined with OR Boolean operators.
2. ‘3D Printing’—the asterisk operation was used for variations of the word ‘print’, accounting for different tenses and all synonyms of 3D printing and 3D were included (Fig. 1).
3. Disease, cancer, injury, trauma, disorder, developmental, and chronic—were all searched for, using the asterisk function to account for all variations.

Within each keyword search ‘OR’ Boolean operators were used to account for all variations of the words. Between keyword groups, ‘AND’ operator was used. Searches changed slightly to account for the format of the database, but overall the same keywords were used (Fig. 1).

### Inclusion and exclusion criteria

Studies were included if they compared patients treated with 3D techniques for MR with patients who received conventional, non-3D aided reconstructions, reporting any outcomes and published from 2011 onwards. 3D printing technology is novel and only started to become more commercially available, including larger cohorts of patients, in the last decade; in order to keep our findings relevant to the current treatments for MR, we only included papers published in the last decade. Title and Abstracts were screened for using the Inclusion Criteria, full-text articles were screened for using the Exclusion Criteria in Table 1; see Fig. 1.

**Table 1:** Inclusion and exclusion criteria used as the screening process for studies identified in our search procedure

Inclusion criteria	Exclusion criteria
Titles and abstracts screening: •Studies published in the last decade, > 2011 •Studies published as articles in Journals •Journals are primarily English speaking (no translations) •Title/Abs contains reference to mandible/mandibular/jaw •Title/Abs contains reference to any form of 3D-printing—additive manufacturing/additive layer manufacturing/rapid prototype modelling/rapid prototyping, computer-aided and computer-assisted manufacturing and manufactured/CAM •Title/Abstract has reference to reconstructive surgery	Full text exclusions: •Focus on dentistry •3D modelling/robotics/virtual planning only (no 3D-printing application) •No specific mandibular reconstruction carried out on patients/non-primary research (systematic and literature reviews) •Non-human/use of cadaver •Non-comparative studies—no comparison between patients undergoing conventional vs. 3D-printing techniques •Clinical disorder necessitating mandibular reconstruction, not specified
Study design—single centre comparative prospective or retrospective studies—including randomized control trials and cohort studies SCImago Journal Rating Q2 or above for the year the paper was published Participants—any patients who had mandibular reconstructions using either 3D methods or conventional methods, as part of the treatment for any clinical disorder Outcomes—we included all outcomes reported in the remaining literature—e.g. operative/ischaemic time, accuracy, complications, aesthetics, cost	

### Patient cohort overlap

There is overlap in the patients treated in both studies by Tarsitano et al. [40, 44]. However both researchers report different outcomes, so have been included in this review.

### Study quality

Journal strength was assessed using the SCImago Journal Rankings. Papers from Journals with a Quartile 2 rating or above were included; the journal rank was based on the year the paper was published.

### Data extraction and presentation

Data were extracted from the remaining 20 papers. This included: study design, year, application of 3D printing technology (Table 2), diagnosis of patients included in the study (Table 3), and outcomes observed (Table 4). Papers were reviewed following PRISMA guidelines [45], as a standardised checklist for evaluating the effectiveness of interventions reported in systematic reviews.

**Table 2:** Summary of the specific 3D-printing techniques used in the studies included in our systematic review

First author, year, country	Title	CAD/CAM techniques used
Ayoub, 2014, Germany [26]	Evaluation of computer-assisted mandibular reconstruction with vascularized iliac crest bone graft compared to conventional surgery: a randomized prospective clinical trial	3D model used to preoperatively contour titanium plates 3D printed cutting/resection guide
Azuma, 2014, Japan [27]	Mandibular reconstruction using plates prebent to fit rapid prototyping 3-dimensional printing models ameliorates contour deformity	3D model used to preoperatively contour titanium plates
Bartier, 2021, France [38]	Computer-assisted versus traditional technique in fibular free-flap mandibular reconstruction: a CT symmetry study	3D model used to preoperatively contour titanium plates 3D printed cutting/resection guide
Ciocca, 2014, Italy [28]	Accuracy of fibular sectioning and insertion into a rapid-prototyped bone plate, for mandibular reconstruction using CAD-CAM technology	3D printed cutting/resection guides Customised patient-specific 3D printed reconstruction plates
De Farias, 2014, Brazil [24]	Use of prototyping in preoperative planning for patients with head and neck tumors	3D model used for preoperative ‘dry run’ surgery 3D model used to preoperatively contour titanium plates
De Maesschalck, 2017, Switzerland [29]	Computer-assisted versus traditional freehand technique in fibular free flap mandibular reconstruction: a morphological comparative study	3D printed cutting/resection guides Customised patient-specific 3D printed reconstruction plates
Gil, 2014, Spain [45]	Surgical planning and microvascular reconstruction of the mandible with a fibular flap using computer-aided design, rapid prototype modelling, and precontoured titanium reconstruction plates: a prospective study	3D model used to preoperatively contour titanium plates 3D printed cutting/resection guides
King, 2018, United States of America [25]	On-site 3-dimensional printing and preoperative adaptation decrease operative time for mandibular fracture repair	3D model used for preoperative ‘dry run’ surgery 3D model used to preoperatively contour titanium plates
Liu, 2014, China [48]	Technical procedures for template-guided surgery for mandibular reconstruction based on digital design and manufacturing	3D model used for preoperative ‘dry run’ surgery 3D model used to preoperatively contour titanium plates
Mahendru, 2020, India [46]	CAD-CAM vs. conventional technique for mandibular reconstruction with free fibula flap: a comparison of outcomes	3D model used to preoperatively contour titanium plates 3D printed cutting/resection guides
Modabber, 2012, Germany [41]	Computer-assisted mandibular reconstruction with vascularized iliac crest bone graft	3D printed cutting/resection guides
Modabber, 2012, Germany [50]	Evaluation of computer-assisted jaw reconstruction with free vascularized fibular flap compared to conventional surgery: a clinical pilot study	3D printed cutting/resection guides 3D model used as a back up for graft shaping
Naros, 2018, Germany [51]	Three-dimensional accuracy of mandibular reconstruction by patient-specific pre-bent reconstruction plates using an “in-house” 3D-printer	3D model used to preoperatively contour titanium plates
Ramanathan, 2020, India [40]	3D planning in mandibular fractures using CAD/CAM surgical splints—a prospective randomized controlled clinical trial	Patient-specific 3D printed occlusal splints
Tarsitano, 2016, Italy [43]	Is a computer-assisted design and computer-assisted manufacturing method for mandibular reconstruction economically viable?	3D printed cutting/resection guide Customised patient-specific 3D printed reconstruction plates
Tarsitano, 2016, Italy [39]	Morphological results of customized microvascular mandibular reconstruction: a comparative study	3D printed cutting/resection guide Customised patient-specific 3D printed reconstruction plates
Wurm, 2019, Germany [52]	The fitting accuracy of pre-bend reconstruction plates and their impact on the temporomandibular joint	3D model used to preoperatively contour titanium plates
Yang, 2021, China [49]	Three-dimensionally printed patient-specific surgical plates increase accuracy of oncologic head and neck reconstruction versus conventional surgical plates: a comparative study	3D printed cutting/resection guide Customised patient-specific 3D printed reconstruction plates
Zhang, 2011, China [47]	Application of rapid prototyping for temporomandibular joint reconstruction	3D model used for preoperative ‘dry run’ surgery 3D model used to preoperatively contour titanium plates
Zhang, 2016, China [53]	Improving the accuracy of mandibular reconstruction with vascularized iliac crest flap: role of computer-assisted techniques	3D model used to preoperatively contour titanium plates 3D printed cutting/resection guides

**Table 3:** Summary of the clinical characteristic of the patients included in our systematic review

First author, year, country	Title	No. of patients	Patient diagnosis	Our categorisation of clinical characteristics^a^
Ayoub, 2014, Germany [26]	Evaluation of computer-assisted mandibular reconstruction with vascularized iliac crest bone graft compared to conventional surgery: a randomized prospective clinical trial	Control: 10 3D group: 10	Control: Osteomyelitis *n* = 4 Keratocyst *n* = 1 SCC *n* = 2 Osteoradionecrosis *n* = 1 Ameloblastoma *n* = 1 Osteonecrosis *n* = 1 3D group: Ameloblastoma *n* = 3 SCC *n* = 3 Osteonecrosis *n* = 1 Osteomyelitis *n* = 2 Ewing sarcoma *n* = 1	Malignant of benign tumour removal Clinical disorder Disease/infection
Azuma, 2014, Japan [27]	Mandibular reconstruction using plates prebent to fit rapid prototyping 3-dimensional printing models ameliorates contour deformity	Control group: 16 3D group: 12	Control: SCC *n* = 15 Osteosarcoma *n* = 1 3D group: SCC *n* = 12	Malignant of benign tumour removal
Bartier, 2021, France [38]	Computer-assisted versus traditional technique in fibular free-flap mandibular reconstruction: a CT symmetry study	Control: 8 3D group: 25	Control: SCC *n* = 3 Osteoradionecrosis *n* = 3 Ameloblastoma *n* = 1 3D group: SCC *n* = 14 Osteoradionecrosis *n* = 7 Ameloblastoma *n* = 2 Sarcoma *n* = 1 *Note: numbers do not add up*	Malignant of benign tumour removal Clinical disorder
Ciocca, 2014, Italy [28]	Accuracy of fibular sectioning and insertion into a rapid-prototyped bone plate, for mandibular reconstruction using CAD-CAM technology	Control: 5 3D group: 5	Control: SCC *n* = 4 Ameloblastoma *n* = 1 3D group: SCC *n* = 2 Ameloblastoma *n* = 2 Osteogenic sarcoma *n* = 1	Malignant of benign tumour removal
De Farias, 2014, Brazil [24]	Use of prototyping in preoperative planning for patients with head and neck tumors	Control: 20 3D group: 17	*Benign and malignant mandibular tumours—numbers not specified*	Malignant of benign tumour removal
De Maesschalck, 2017, Switzerland [29]	Computer-assisted versus traditional freehand technique in fibular free flap mandibular reconstruction: a morphological comparative study	Control: 11 3D group: 7	Control: SCC *n* = 6 Osteoradionecrosis *n* = 5 3D group: SCC *n* = 6 Osteoradionecrosis *n* = 1	Malignant of benign tumour removal Clinical disorder
Gil, 2014, Spain [45]	Surgical planning and microvascular reconstruction of the mandible with a fibular flap using computer-aided design, rapid prototype modelling, and precontoured titanium reconstruction plates: a prospective study	Control: 10 3D group: 10	Control: SCC *n* = 8 Osteoradionecrosis *n* = 1 Osteosarcoma *n* = 1 3D group: SCC *n* = 4 Osteomyelitis *n* = 2 Osteoradionecrosis *n* = 2 Ameloblastoma *n* = 2	Malignant of benign tumour removal Clinical disorder Disease/infection
King, 2018, United States of America [25]	On-site 3-dimensional printing and preoperative adaptation decrease operative time for mandibular fracture repair	Control: 19 3D group: 19	Control: fracture *n* = 19 3D group: fracture *n* = 19	Injury/trauma
Liu, 2014, China [48]	Technical procedures for template-guided surgery for mandibular reconstruction based on digital design and manufacturing	Control: 8 3D group: 15	Control: not specified 3D group: Ameloblastoma *n* = 7 Fibroma *n* = 4 Gingival carcinoma *n* = 4	Malignant of benign tumour removal
Mahendru, 2020, India [46]	CAD-CAM vs. conventional technique for mandibular reconstruction with free fibula flap: a comparison of outcomes	Control: 40 3D group: 40	Control: SCC *n* = 36 Ameloblastoma *n* = 4 3D group: SCC *n* = 37 Ameloblastoma *n* = 3	Malignant of benign tumour removal
Modabber, 2012, Germany [41]	Computer-assisted mandibular reconstruction with vascularized iliac crest bone graft	Control: 15 3D group: 5	Control: SCC *n* = 6 Osteonecrosis *n* = 2 Osteoradionecrosis *n* = 2 Osteomyelitis *n* = 2 Gunshot wound *n* = 1 Ameloblastoma *n* = 1 Keratocyst *n* = 1 3D group: SCC *n* = 2 Ameloblastoma *n* = 1 Osteosarcoma *n* = 1 Pseudoarthrosis *n* = 1	Malignant of benign tumour removal Clinical disorder Disease/infection Injury/trauma
Modabber, 2012, Germany [50]	Evaluation of computer-assisted jaw reconstruction with free vascularized fibular flap compared to conventional surgery: a clinical pilot study	Control: 5 3D group: 5	Control: SCC *n* = 2 Rhabdomyosarcoma *n* = 1 Midfacial projectile *n* = 1 Keratocyst *n* = 1 3D group: SCC *n* = 1 Chondrosarcoma *n* = 1 Ameloblastoma *n* = 1 Midfacial projectile *n* = 2	Malignant of benign tumour removal Injury/trauma
Naros, 2018, Germany [51]	Three-dimensional accuracy of mandibular reconstruction by patient-specific pre-bent reconstruction plates using an “in-house” 3D-printer	Control: 21 3D group: 21	Control: SCC *n* = 19 Cancer (unknown primary) *n* = 1 Ameloblastoma *n* = 1 3D group: SCC *n* = 19 Osteoradionecrosis *n* = 2	Malignant of benign tumour removal Clinical disorder
Ramanathan, 2020, India [40]	3D planning in mandibular fractures using CAD/CAM surgical splints—a prospective randomized controlled clinical trial	Control: 15 3D group: 15	Control: fracture *n* = 15 3D group: fracture *n* = 15	Injury/trauma
Tarsitano, 2016, Italy [43]	Is a computer-assisted design and computer-assisted manufacturing method for mandibular reconstruction economically viable?	Control: 20 3D group: 20	Control: SCC *n* = 14 Ameloblastoma *n* = 3 Osteosarcoma *n* = 1 Keratocyst *n* = 2 3D group: SCC *n* = 12 Ameloblastoma *n* = 4 Osteosarcoma *n* = 2 Keratocyst *n* = 2	Malignant of benign tumour removal
Tarsitano, 2016, Italy [39]	Morphological results of customized microvascular mandibular reconstruction: a comparative study	Control: 30 3D group: 30	*Malignant of benign tumour lesions—not specified*	Malignant of benign tumour removal
Wurm, 2019, Germany [52]	The fitting accuracy of pre-bend reconstruction plates and their impact on the temporomandibular joint	Control: 20 3D group: 20	Control: SCC *n* = 9 Osteoradionecrosis *n* = 6 Ameloblastoma *n* = 2 Other *n* = 3 3D group: SCC *n* = 12 Osteoradionecrosis *n* = 2 Keratocyst *n* = 3 Other *n* = 3	Malignant of benign tumour removal Clinical disorder
Yang, 2021, China [49]	Three-dimensionally printed patient-specific surgical plates increase accuracy of oncologic head and neck reconstruction versus conventional surgical plates: a comparative study	Control: 16 3D group: 17	Control: Benign tumour = 4 Malignant tumour *n* = 11 Other n = 1 3D group: Benign *n* = 3 Malignant *n* = 12 Other *n* = 2 *Specifics not specified*	Malignant of benign tumour removal Clinical disorder
Zhang, 2011, China [47]	Application of rapid prototyping for temporomandibular joint reconstruction	Control: 24 3D group: 11	Control: Condylar osteochondroma *n* = 1 Osteomyelitis *n* = 1 Joint ankylosis *n* = 16 Giant cell tumour *n* = 3 Condylar self-absorption *n* = 1 Ameloblastoma *n* = 1 Osteoradionecrosis *n* = 1 3D group: Acquired deformity *n* = 1 Post-op mandible defect *n* = 1 Osteonecrosis *n* = 2 Ossifying fibroma *n* = 1 joint ankylosis *n* = 1 Osteoarthrosis *n* = 2 Giant cell tumour of bone *n* = 1 Fibrous dysplasia *n* = 1 Ameloblastoma *n* = 1	Malignant of benign tumour removal Clinical disorder Disease/infection
Zhang, 2016, China [53]	Improving the accuracy of mandibular reconstruction with vascularized iliac crest flap: role of computer-assisted techniques	Control: 30 3D group: 15	Control: Ameloblastoma *n* = 15 Ossifying fibroma *n* = 7 Odontogenic myxoma *n* = 3 Odontogenic ghost cell tumour *n* = 1 Gingival carcinoma *n* = 3 Osteosarcoma *n* = 1 3D group: Ameloblastoma *n* = 10 Ossifying fibroma *n* = 5	Malignant of benign tumour removal

*SCC* squamous cell carcinoma

^a^Due to the heterogeneity of the clinical causes included, we grouped these into four categories; Malignant or benign tumour removal, any type of clinical disorder (Osteoradionecrosis, Osteonecrosis, Pseudoarthrosis, acquired deformity, Joint ankylosis, osteoarthrosis, fibrous dysplasia and condylar reabsorption), any type of disease or infection (osteomyelitis), any type of injury or trauma

**Table 4:** Summary of the outcomes observed in the studies included in our systematic review

First author, year, country	Title	Outcomes observed^a^
Ayoub, 2014, Germany [26]	Evaluation of computer-assisted mandibular reconstruction with vascularized iliac crest bone graft compared to conventional surgery: a randomized prospective clinical trial	Operative/ischaemic time ^b^Accuracy—bone harvested/bone used ^b^Accuracy—pre- and postoperative measurements Cost
Azuma, 2014, Japan [27]	Mandibular reconstruction using plates prebent to fit rapid prototyping 3-dimensional printing models ameliorates contour deformity	^b^Accuracy—postoperative bilateral measurements
Bartier, 2021, France [38]	Computer-assisted versus traditional technique in fibular free-flap mandibular reconstruction: a CT symmetry study	Accuracy—pre- and postoperative Measurements
Ciocca, 2014, Italy [28]	Accuracy of fibular sectioning and insertion into a rapid-prototyped bone plate, for mandibular reconstruction using CAD-CAM technology	Accuracy—pre- and postoperative measurements Cost
De Farias, 2014, Brazil [24]	Use of prototyping in preoperative planning for patients with head and neck tumors	Operative/ischaemic time Accuracy—bone harvested/bone used Aesthetic outcome
De Maesschalck, 2017, Switzerland [29]	Computer-assisted versus traditional freehand technique in fibular free flap mandibular reconstruction: a morphological comparative study	Accuracy—pre- and postoperative measurements
Gil, 2014, Spain [45]	Surgical planning and microvascular reconstruction of the mandible with a fibular flap using computer-aided design, rapid prototype modelling, and precontoured titanium reconstruction plates: a prospective study	Operative/ischaemic time Incidence of postoperative complications
King, 2018, United States of America [25]	On-site 3-dimensional printing and preoperative adaptation decrease operative time for mandibular fracture repair	Operative/ischaemic time Cost
Liu, 2014, China [48]	Technical procedures for template-guided surgery for mandibular reconstruction based on digital design and manufacturing	Operative/ischaemic time
Mahendru, 2020, India [46]	CAD-CAM vs. conventional technique for mandibular reconstruction with free fibula flap: a comparison of outcomes	Operative/ischaemic time Incidence of postoperative complications Aesthetic outcome
Modabber, 2012, Germany [41]	Computer-assisted mandibular reconstruction with vascularized iliac crest bone graft	Operative/ischaemic time Accuracy—bone harvested/bone used Aesthetic outcome
Modabber, 2012, Germany [50]	Evaluation of computer-assisted jaw reconstruction with free vascularized fibular flap compared to conventional surgery: a clinical pilot study	Operative/ischaemic time Accuracy—bone harvested/bone used
Naros, 2018, Germany [51]	Three-dimensional accuracy of mandibular reconstruction by patient-specific pre-bent reconstruction plates using an “in-house” 3D-printer	Accuracy—postoperative bilateral measurements
Ramanathan, 2020, India [40]	3D planning in mandibular fractures using CAD/CAM surgical splints—a prospective randomized controlled clinical trial	Incidence of postoperative complications Aesthetic outcome
Tarsitano, 2016, Italy [43]	Is a computer-assisted design and computer-assisted manufacturing method for mandibular reconstruction economically viable?	Operative/ischaemic time Incidence of postoperative complications Cost
Tarsitano, 2016, Italy [39]	Morphological results of customized microvascular mandibular reconstruction: a comparative study	Accuracy—pre- and postoperative measurements
Wurm, 2019, Germany [52]	The fitting accuracy of pre-bend reconstruction plates and their impact on the temporomandibular joint	Accuracy—postoperative bilateral measurements
Yang, 2021, China [49]	Three-dimensionally printed patient-specific surgical plates increase accuracy of oncologic head and neck reconstruction versus conventional surgical plates: a comparative study	Operative/ischaemic time Accuracy—postoperative bilateral measurements Accuracy—pre- and postoperative measurements Cost
Zhang, 2011, China [47]	Application of rapid prototyping for temporomandibular joint reconstruction	Operative/ischaemic time Accuracy—postoperative bilateral measurements
Zhang, 2016, China [53]	Improving the accuracy of mandibular reconstruction with vascularized iliac crest flap: role of computer-assisted techniques	Accuracy—pre- and postoperative measurements Incidence of postoperative complications

^a^Due to heterogeneity in the reporting of outcomes observed we groups our findings into five categories: operative/ischaemic time (*n* = 11), accuracy (*n* = 16), incidence of postoperative complications (*n* = 5), assessment of aesthetic outcome (*n* = 3) and cost (*n* = 5)

^b^Accuracy itself was also very heterogenous, based on our findings we further categorised this into three categories: bone harvested vs. bone used (*n* = 4), postoperative bilateral measurements (*n* = 5) and comparison between pre- and postoperative CT scans (*n* = 7)

## Data Availability

The datasets used and/or analysed during the current study are available from the corresponding author on reasonable request.

## Abbreviations

3D: Three-dimensional
CAD/CAM: Computer-aided design and computer-aided manufacturing
CT: Computed tomography
DCIA: Deep circumflex iliac artery
DICOM: Digital Imaging and Communications in Medicine
FFF: Fibula free flap
MR: Mandibular reconstruction
SOFF: Scapular osteocutaneous free flap
RP: Rapid prototyping
